# Study of Transition Areas in Press-Hardened Steels in a Combined Tool for Hot and Cold Forming

**DOI:** 10.3390/ma16010442

**Published:** 2023-01-03

**Authors:** Filip Votava, Hana Jirková, Ludmila Kučerová, Štěpán Jeníček

**Affiliations:** Regional Technological Institute, University of West Bohemia, Univerzitní 8, 301 00 Pilsen, Czech Republic

**Keywords:** press-hardening, hot stamping, high-strength steel, multiphase structure

## Abstract

Press-hardening, also known as hot stamping, is a manufacturing process for producing car body parts that must meet the high demands of their mechanical properties and safety parameters. Moreover, these components often require different mechanical properties in different parts of the component. This work presents the press-hardening process in a special combined tool where one half of the tool is heated and the other half is cooled. The cooled part has been 3D printed due to the complexity of the internal cooling channels. The aim of this work is to investigate the variation of the microstructures in the sheet metal and the mechanical properties in relation to the cooling process in the tool and to determine the transition area where these properties cross over. Two steels were chosen for the experiment. The most commonly used steel 22MnB5, and an experimental high-strength steel with 0.2% C alloyed with manganese and aluminium. A temperature of 425 °C was set in the heated part of the tool, and different holding times in the tool were tested. In the heated part of the tool, a bainitic structure with a fraction of ferrite and retained austenite was formed, while in the quenched part of the tool, a martensitic transformation was promoted due to rapid cooling. In addition to microscopic analyses, mechanical tests and hardness measurements were also performed.

## 1. Introduction

**Figure 1:**
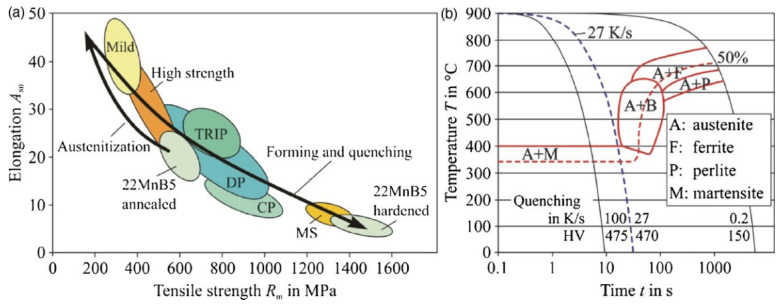
(**a**) Mechanical properties of 22MnB5 and (**b**) CCT diagram [3].

The use of ultrahigh-strength steels (UHSS) is still rapidly increasing in the automotive industry. This is mainly because of the improvement in the results obtained for the material from crash safety tests. It is related to the increase in mechanical properties and the reduction of vehicle weight. UHSS are used in the hot stamping process for parts such as side impact reinforcement beams, A-pillars, B-pillars, and bumpers [1,2]. The entire automotive industry is trying to achieve as much sustainability as possible. A good example is the large expansion of electric car production in order to reduce CO_2_ emissions. Reducing the weight of vehicle parts can lead directly to a reduction in fuel consumption, and therefore to a reduction in the quantity of exhaust gases produced from internal combustion and diesel engines, and in the case of electric cars to a reduction in electricity consumption [3,4]. New car models are at least 20% lighter than old models [1,5]. The highest use of hot-formed steels is in the Volvo XC (2nd generation since 2014) where 38% of the body mass of all the steels used are hot formed [1]. The hot stamping process enables the production of these lighter parts with excellent ultimate tensile strength (UTS), up to around 1500 MPa [6]. Press hardening was initially developed for boron steels. The best-known representative is 22MnB5 steel which can produce a martensitic microstructure that is important for enhancing the side crash performance in B-pillar parts [7,8]. With 22MnB5 steel, it is necessary to reach a cooling rate of at least 27 °C/s, as shown in Figure 1, (possibly up to 30 °C/s) to gain martensitic microstructures [3,9,10,11].

Hot stamping has a number of advantages over conventional stamping at room temperature. The main advantages are the need for lower forming forces, a decrease in spring back, and simultaneous control of complex microstructures during thermomechanical processing. This means it is possible to vary mechanical properties and combinations using a single blank. These hot-stamped parts with tailored properties can avoid particular problems, such as two different materials being welded together, the presence of a weld, and heat-affected zones (HAZ), and during crash test conditions when using a material with local soft areas. Moreover, complex geometries with different thicknesses and strength levels can be manufactured by using tailored blanks in hot stamping [12,13]. Several articles have been written that discuss different ways of making combined tools (i.e., partially heated and partially cooled). The first option for manufacturing a combined tool is to drill in multiple directions to create cooling channels in the tool then plugging some of the holes. A drilled tool for aluminium sheet processing was prepared by Meza-García et al. [14]. Hoffmann et al. also used drilling to create cooling channels and optimized them. The diameters of the holes ranged from 8 to 16 mm [15]. Schieck et al. evaluated the advantages and disadvantages of drilled cooling channels, shell design, and cast-in cooling channels. For example, drilled tools have a good cooling ability, but more complex geometries cannot be produced. Cast-in cooling channels are cost-effective to make, suitable for complicated shapes, and there are no sealing issues as in the case of drilled tools. On the other hand, they have to be made of cast iron, are difficult to repair if broken, and have a lower cooling capacity [16].

After considering the advantages and disadvantages, it was decided to use the most modern variant of additive manufacturing, direct metal laser sintering (DMLS), which has almost no limits in terms of cooling channel design. Moreover, we decided to produce a half-heated and half-cooled tool, i.e., a combined tool suitable for the production of tailored sheets. In the tailored tempering process (TTP) of 22MnB5, or tailored hot stamping (THS), different distributions of mechanical properties in the same component can be achieved [17]. Conventional hot stamping requires the blank to be heated up to the austenitizing temperature and transferred to a cooled stamping die for forming and quenching. There are two types of phase transformation, austenitisation and cooling transformations of austenite, which occur during the stamping procedure. Tailored partial cooling and partial heating process variants are designed according to this special feature of hot stamping to obtain parts with tailored properties [18].

The production of a combined tool for experimental purposes can provide new in-sights into the field of hot stamping, both in terms of determining the optimal setup of the process parameters and in terms of using new materials suitable for this processing method.

## 2. Experimental Programme

### 2.1. Materials

Two materials were used for the experiment. The first material was 22MnB5 steel with a thickness of 1.5 mm and the chemical composition shown in Table 1, which was used to verify the functionality of the tool and also as a standard to compare the results with other materials that will be processed in the future. The second material was a sheet of 20MnAl experimental steel with a thickness of 1.7 mm for hot stamping, which is expected to produce tailored properties and in addition, could take advantage of complex multi-phase microstructures. The chemical composition of the ingot is shown in Table 1. Since this is experimental steel without a standardized marking, the marking for this steel was created based on its chemical composition, with the marking similar to the 22MnB5 steel. The ingot was forged, hot rolled, annealed, machined, and cold rolled to a final thickness of 1.8 ± 0.05 mm. This indicates that the sheets had to be ground to a thickness of 1.7 mm before use. For the experiment, 240 mm × 210 mm specimens were prepared from sheet metal.

### 2.2. Combined Tool

**Figure 2:**
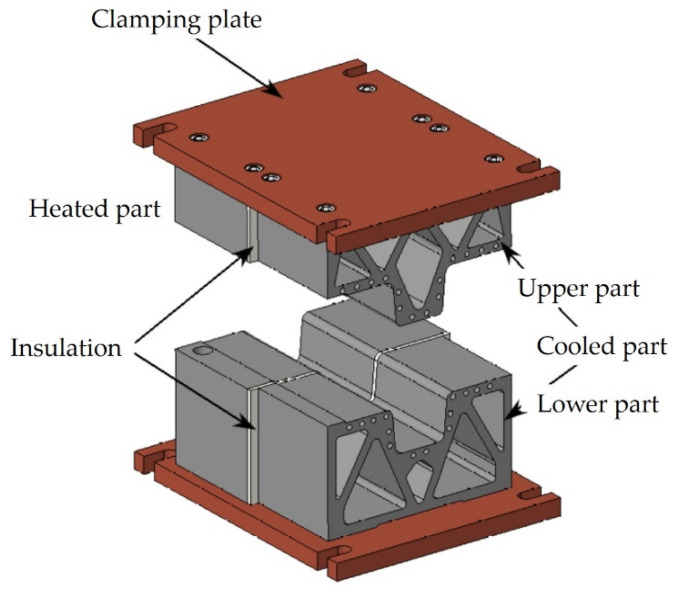
CAD assembly of the combined tool.

**Figure 3:**
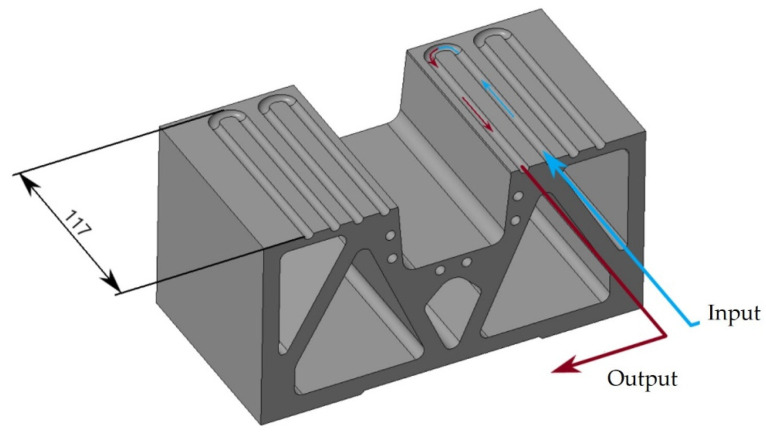
Cut view of the cooling channels in the female part of the tool.

A combined hot stamping tool was used for the experiment. Figure 2 shows the CAD model of the tool. The heated part has holes for HPCH (i.e., high power cartridge heater) stainless steel heating cartridges with a diameter of 20 mm and a length of 125 mm, capable of reaching temperatures of up to 750 °C. The upper part has three cartridges and the lower has four cartridges. All the cartridges are connected to a heating source with PID controllers. Figure 3 shows the detail of the cooled part of the tool with the cut view of the cooling channels inside. The tool was printed from MS1 material on the EOS M290 3D printer which works on the principle of DMLS (direct metal laser sintering). The models underwent topological optimization to save material. Heat treatment in a protective argon atmosphere was carried out after printing to reduce internal stresses. It consisted of heating to 820 °C with a dwell time of one hour and aging at 490 °C for six hours [19]. Hoses with threaded brass hose tails were connected to the cooled part. The cooling medium was water which flowed through distribution blocks made of aluminium alloy, one with 14 inputs of water and the other with 14 outputs. Between the heated and cooled parts there were insulating plates made of laminate material PAMITHERM 41,130 with a thickness of 5 mm.

**Figure 4:**
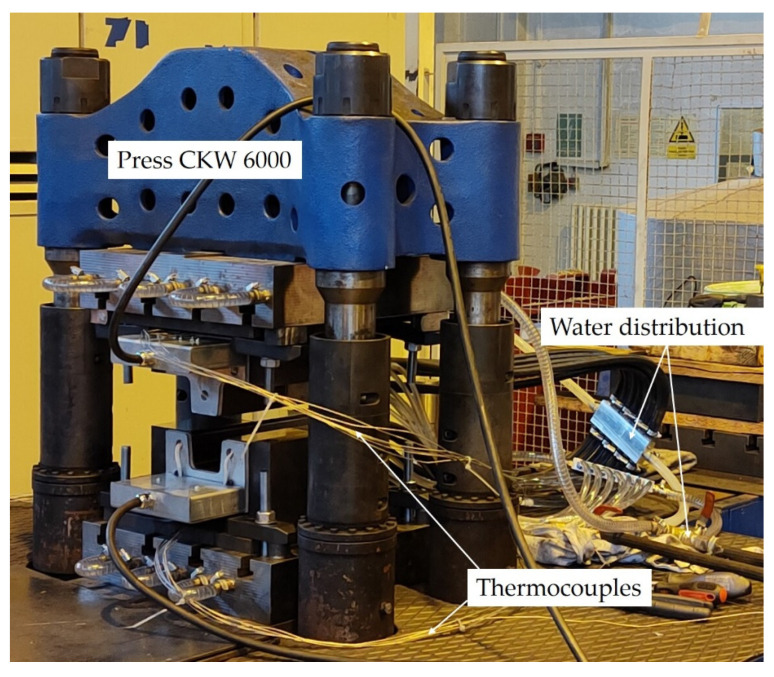
Workplace with the combined tool including all the accessories.

There were 12 holes in the heated part for mounting K-type insulated thermocouples with a diameter of 1.5 mm which controlled the temperature during the hot stamping process. One thermocouple from both heated parts was connected to a heating source with PID controllers which then regulated the set temperature of the heating cartridges. The other 10 thermocouples were connected to the data logger, which captured temperatures from various locations in the tool during the hot stamping process. Figure 4 shows the set-up of the workplace with the combined tool placed in a CKW 6000 hydraulic forging press.

### 2.3. Different Heat Treatment Parameters

**Figure 5:**
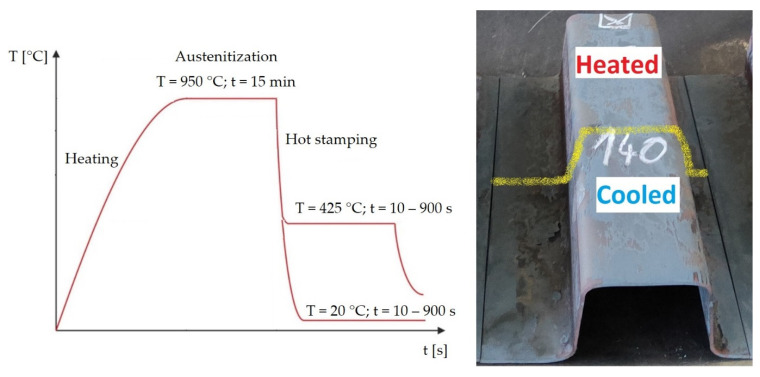
Heating curves for both parts of the sheet and the final shape of the omega profile.

A laboratory electric chamber resistance furnace LAC LH 30/13 was used to heat the sheets. Austenitisation was carried out at 950 °C. A soaking time of 15 min was chosen. This may seem excessive if we consider the results of [20], where minimum times are defined for given thicknesses of 22MnB5 sheets so that a hardness of 470 HV10 is achieved after quenching in the tool. For a thickness of 1.5 mm, 2.75 min is given in the paper. For a thickness of 1.75 mm at 950 °C it is 3 min. The longer time in our experiment was chosen based on previous experience with austenitizing times in this furnace for sheets with smaller dimensions for forming with a smaller hot stamping tool. The choice for the heated parts of the tool was based on the fact that the temperature of 425 °C is also above the Ms temperature for 22MnB5 [9]. Min et al. reported a Ms value of 411 °C [21]. In addition, using JMatPro software (version 12.1), the temperatures of Ms and A3 were calculated to be 369.5 °C and 813.9 °C, respectively. For the 20MnAl material, the Ms temperature was 428.3 °C and the A3 temperature was 993.1 °C. These parameters were kept for both experimental materials in order to compare the properties of the materials. The cooled part had a temperature of approximately 20 °C, where the cooling is controlled by the water flow rate. The selected dwell times of the sheet in the tool were graded as follows: 10 s; then subsequent steps with a dwell time progressively increasing by 1 min, i.e., 70; 140; 210 s; with the final time of 900 s (15 min). The summary data of the experimental parameters are shown in Table 2. Figure 5 shows the processed sheet with the cooling curves for the parts.

The transfer between the furnace and the tool took approximately 5 s, the tool closing took 2 s. The press speed was 100 mm/s. After the specified time in the tool, the sheet was removed and further cooled freely in air.

### 2.4. Equipment Used for Evaluation

The sampling for metallography was always carried out from three locations on the sheet: cooled (C), transition area (T), and heated (H). The samples were cut with a water jet to avoid creating a thermally affected area. The samples for the mini tensile test were then taken by electro-erosive machining and ground to the required thickness, i.e., 1.2 mm. In all cases, the samples were taken from the cooled and heated parts.

Preparation of the samples for metallography consisted of grinding on sandpaper with grits ranging from 240 to 1200. This was followed by polishing on diamond slurry screens with grits of 3 and 1 µm. The etching was carried out in a Nital etchant (3%). The samples were observed on an Olympus light microscope (Tokyo, Japan). Scanning electron microscopy (SEM) Tescan VEGA 3 (Brno, Czech Republic) for 22MnB5 material and Zeiss Crossbeam 340-47-44 (Oberkochen, Germany) for 20MnAl material were used for detailed microstructure analysis. For verification of the amount of retained austenite, XRD measurements were taken in a Panalytical X’Pert PRO diffractometer with Co Kα radiation (0.1789 nm) and an X’celerator detector (Malvern, UK). The patterns were collected in the range of 30–130 degrees, with a step of 0.05 deg. They were subsequently processed by X’Pert HighScore Plus using PDF4 database software to perform phase identification. Finally, Rietveld refinements were performed employing TOPAS V3 software to determine the quantification of phase composition. In addition, Vickers hardness measurements (LECO Instrumente Plzeň, spol. s r. o., Pilsen, Czech Republic) were performed with a load of 10 kg. In the transition region, the hardness profile was measured for the 20MnAl material with a load of 1 kg and an indentation spacing of 0.3 mm over a total path length of 12 mm for the limiting times (10 and 900 s).

The tensile test was performed according to ČSN EN ISO 6892-1 method A on a ZWICK Roel Z250 machine (Ulm, Germany) on mini tensile specimens with an active length of 5 mm and a cross-section of 2 mm × 1.2 mm. The values given are the average of two tensile tests.

## 3. Results

**Figure 6:**
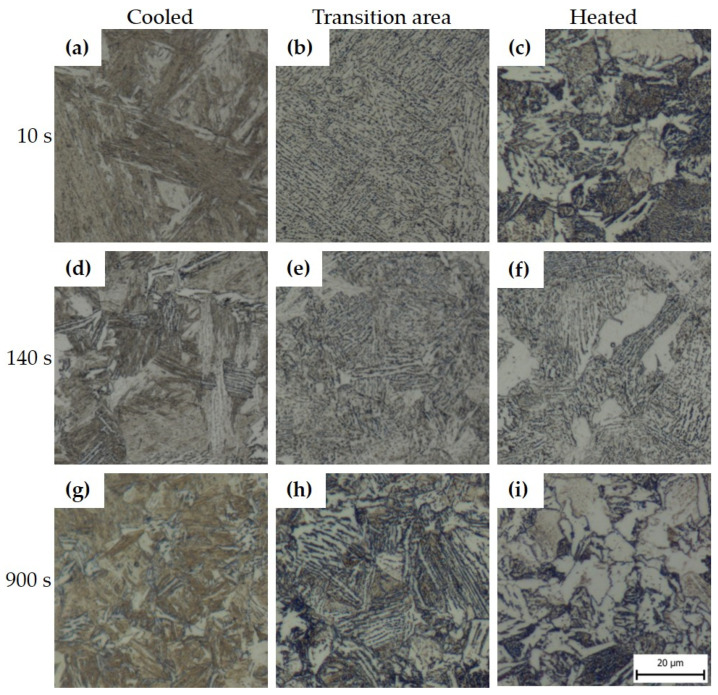
Microstructures of 22MnB5 after hot stamping seen under an optical microscope: (**a**) 10s_C, (**b**) 10s_T, (**c**) 10s_H, (**d**) 140s_C, (**e**) 140s_T, (**f**) 140s_H, (**g**) 900s_C, (**h**) 900s_T, (**i**) 900s_H.

It is visible in the light microscopy photos that the different tool dwell times affected the resulting structure. Figure 6 shows the images of 22MnB5 for each region of the omega profile. Images were selected for the limiting dwell times (i.e., 10 s and 900 s) and 140 s. In the cooled part of the tool, the microstructure was predominantly martensite in the case of the shortest dwell time of 10 s (Figure 6a) or martensitic–bainitic with a proportion of bainite increasing with increasing dwell time (Figure 6d,g). In the transition area between the cooled and heated parts of the tool, the microstructure changed. For the shorter dwell times in the tool in Figure 6b,e, the structure was mainly composed of lamellar bainite and a small proportion of martensite. At the longest dwell time (i.e., 900 s), pearlite was detected in the structure in addition to bainite. In the part of the tool heated to 425 °C, the influence of a different cooling rate profile compared to the cooled part was evident. Due to the cooling stopping at 425 °C, which is above the Ms temperature for this steel, only a small amount of fine martensite was formed after removing the sheet from the tool. In the case of the shortest tool dwell time of 10 s, the structure was mainly composed of pearlite and ferrite, as well as in the longest dwell time of 900 s (Figure 6c,i). A different character of the structure was obtained for tool dwell times of 70, 140, and 210 s, where a multiphase structure consisting of ferrite, bainite, and pearlite was obtained. The presence of large coarse blocks of lamellar bainite at the dwell time of 140 s was due to the suitable dwell time for its formation (Figure 6f). In addition, areas of pearlite and martensite are not visible. The results of XRD measurements of retained austenite showed in all cases a result below the detection limit of the method, the only exception was sample 10s_H where a value of 5 ± 1 wt. % was measured.

**Figure 7:**
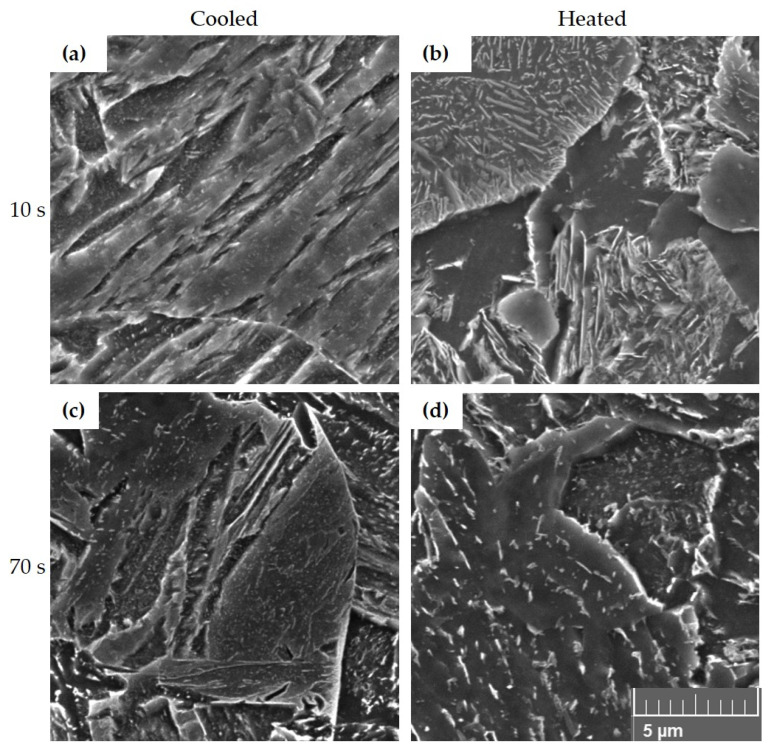
Microstructures of 22MnB5 after hot stamping under a scanning electron microscope: (**a**) 10s_C, (**b**) 10s_H, (**c**) 70s_C, (**d**) 70s_H.

For a more detailed analysis, the microstructures were also documented on a Tescan VEGA 3 scanning electron microscope (Figure 7). For the SEM images of 22MnB5 steel, only samples with tool dwell times of 10 and 70 s were selected because these times should be sufficient to achieve martensite in the structure. It is clear from the microstructure images that at a tool dwell time of 10 s in the cooled part, the structure was predominantly martensite, while at the same time, significant precipitation of very fine particles can be seen following the martensitic needles (Figure 7a). The presence of lamellar pearlite was confirmed in the heated part (Figure 7b). During processing with a tool dwell time of 70 s, a martensitic structure was observed in the cooled part of the tool with a proportion of lower bainite and precipitations along the boundaries and within the martensitic and bainitic formations (Figure 7c). In the heated part, rather coarser lower-bainite blocks were observed with significant precipitation along the bainitic ferrite laths, occasional ferritic grains, and rather rare small pearlitic islands (Figure 7d).

**Figure 8:**
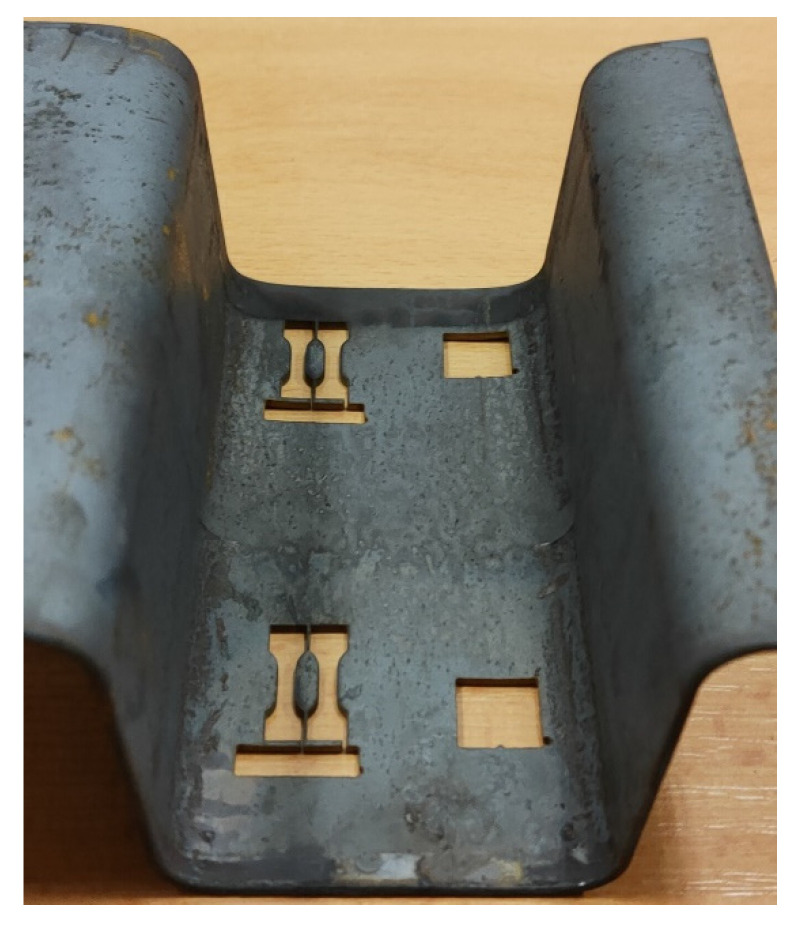
Position of mini tensile specimens on the omega profile product from cooled and heated part.

**Figure 9:**
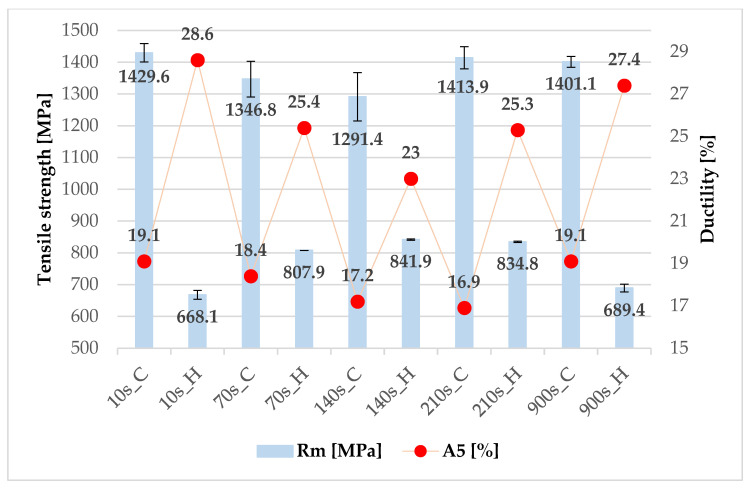
Mechanical properties of 22MnB5 steel (cooled (C) and heated (H) part).

Two samples per area were also taken from the cooled and heated areas of the omega profiles for mini tensile tests, shown in Figure 8. The resulting values are shown in Figure 9. We can observe a trend of high ultimate tensile strength (R_m_) for the cooled section with a lower ductility value (A_5_). On the other hand, for the heated part, the ductility is higher and the ultimate tensile strength is lower. The measured values of R_m_ are consistent with the findings of other papers, however, the values of ductility are noticeably higher. This is due to the size of the specimens used for the tensile test. A similar issue of affected ductility in terms of geometry was reported by Jirková et al. [22] and Opatová et al. [23]. The results are then only comparable mainly between each other, or with specimens with a similar length for the active part.

The results show that in the cooled part of the tool an ultimate tensile strength limit of over 1300 MPa was achieved for all processing parameters. To achieve a higher ultimate tensile strength limit of 800 MPa in the heated part, it was necessary to attain a bainitic structure. This was observed mainly for dwell times from 70 to 210 s. When a ferritic-pearlitic structure was obtained, the ultimate tensile strength limit dropped to values around 680 MPa (10 and 900 s dwell time). Cooling in the heated part of the tool had an influence on the ductility value. The slower cooling rate, the dwell at 425 °C, and the absence of martensite in the structure led to an increase in ductility to values between 23 and 28.6%.

Table 3 shows the HV10 hardness values with standard deviation. In 22MnB5 there was a decrease in hardness from the cooled part to the heated part. At 10 s dwell, 469 ± 7 HV10 was achieved in the cooled part, 254 ± 2 HV10 in the transition area, and 216 ± 4 HV10 in the heated part.

**Figure 10:**
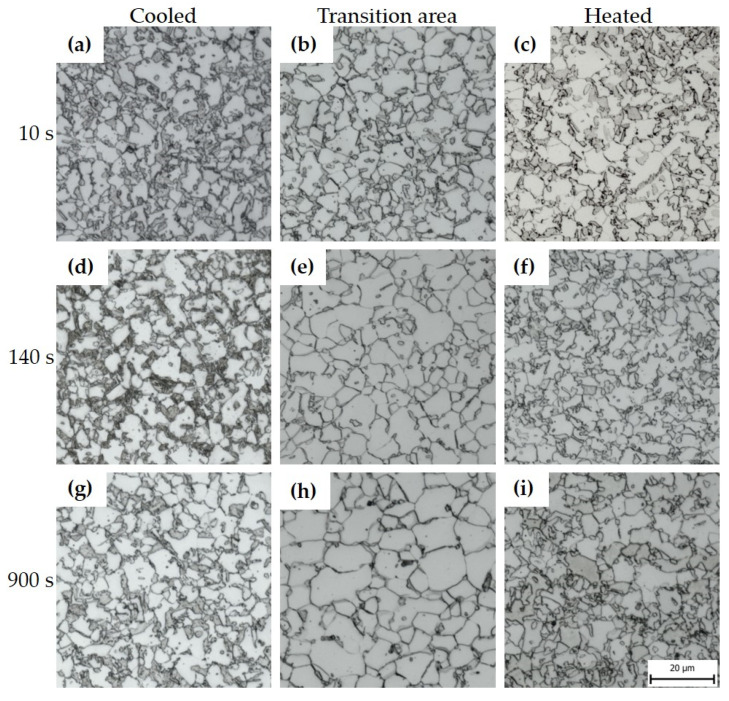
Microstructures of 20MnAl after hot stamping under an optical microscope: (**a**) 10s_C, (**b**) 10s_T, (**c**) 10s_H, (**d**) 140s_C, (**e**) 140s_T, (**f**) 140s_H, (**g**) 900s_C, (**h**) 900s_T, (**i**) 900s_H.

For 20MnAl steel, the structure in the cooled part of the tool was a combination of proeutectoid ferrite, martensite, and a small proportion of retained austenite or so-called M-A constituent, i.e., partially transformed islands of residual austenite to martensite (Figure 10a,d,g). A small amount of bainite was also detected in the structure. Retained austenite was present along the margins of the martensitic islands in M-A constituents as well as between the bainitic ferrite lamellae. The bainite was carbide-free in all samples of 20MnAl steel, consisting of a mixture of bainitic ferrite laths and retained austenite (or M-A constituents) laths or islands. This morphology of bainite is typical for advanced high strength low alloyed steels with chemical compositions similar to 20MnAl steel [24]. A significant decrease in the proportion of martensite was found in the transition area of the tool for all analysed samples. However, increasing ferrite phase fraction and ferrite grain coarsening were observed with increasing dwell time as is apparent from the comparison of Figure 10b,h. When the forming was carried out in the heated part of the tool with a dwell time of 10 s, the structure consisted of proeutectoid ferrite, bainite, and a large proportion of the so-called M-A constituent, i.e., partially transformed islands of residual austenite to martensite. At longer dwell times of 70 and 140 s, the austenitic islands were more stabilized and less transformed to martensite during the final cooling after removal of the profile from the tool, and the proportion of martensite decreased. This was reflected by the results of the retained austenite quantification by XRD measurements of the heated parts. While the amount of austenite in sample 10s_C was below detection limit of the method, 9 ± 2 wt. % of retained austenite were found in 210s_C sample, and 8 ± 2 wt. %. in 900s_C. At the longest tool dwell time of 900 s, large grains of M-A constituent partially transformed to martensite were detected in the structure.

**Figure 11:**
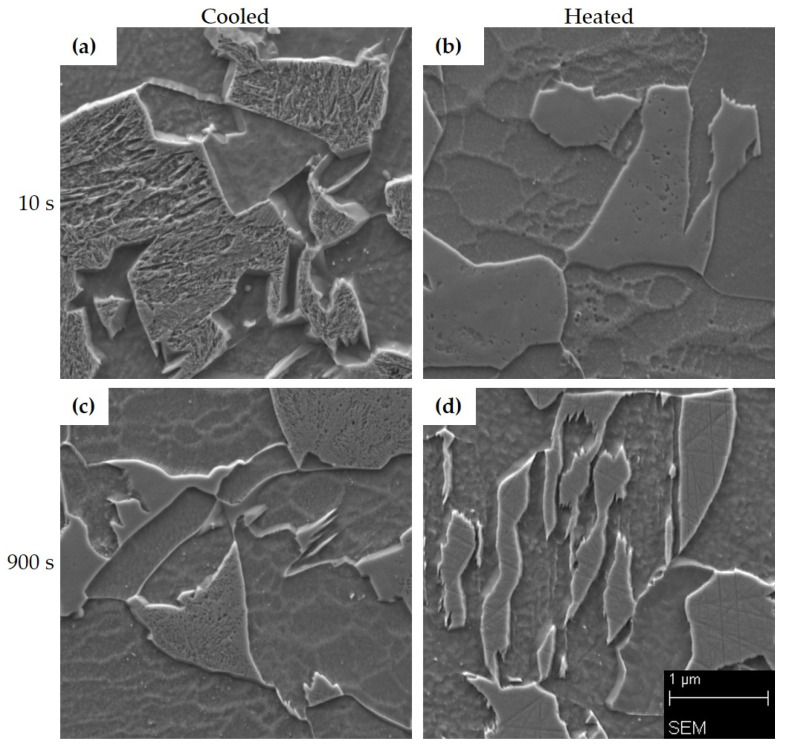
Microstructures of 20MnAl after hot stamping under a scanning electron microscope: (**a**) 10s_C, (**b**) 10s_H, (**c**) 900s_C, (**d**) 900s_H.

The morphology of martensite and bainite was observed on a Zeiss Crossbeam 340-47-44 scanning electron microscope (Figure 11). Figure 11a shows the detail of a martensitic islands surrounded by ferritic grains. A very similar character of the microstructure was confirmed in the heated part as well (Figure 11b), even though the martensitic (or M-A constituent) islands in Figure 11b look much smoother than in Figure 11a and only groups of fine dimples are suggesting the occurrence of the martensite. The identification of the islands as martensitic is further supported by the results of XRD phase diffraction analysis which did not detect a significant amount of retained austenite in this microstructure, and also the hardness values were similar to those of the cooled part. With a dwell time of 900 s, untransformed islands of residual austenite were detected in the structure (Figure 11c,d) as well as islands of partially transformed M-A constituent (Figure 11c).

**Figure 12:**
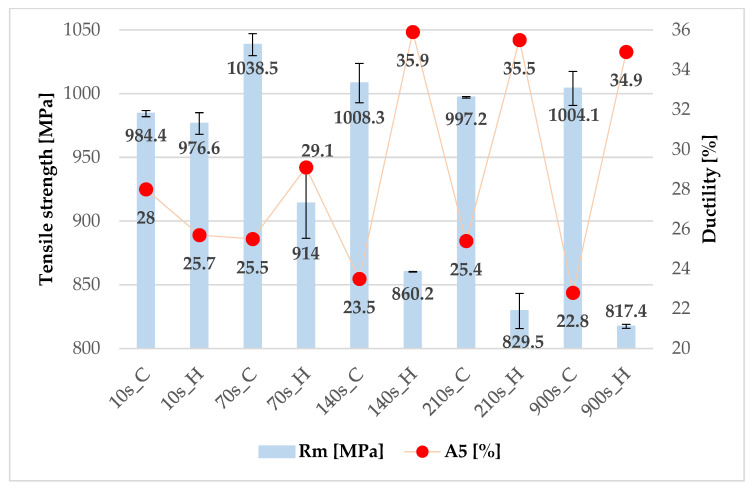
Mechanical properties for 20MnAl steel (cooled (C) and heated (H) part).

The results from the mini tensile strength tests of 20MnAl steel (Figure 12) correspond to the microstructures. For the shortest tool dwell time of 10 s, very similar values of ultimate strength of about 980 MPa and ductility in the range of 26 to 28% were obtained. These results show that the cooling rate, which varied in the different parts of the tool, did not have a significant effect on the evolution of the structure and, due to the short dwell time, there was no stabilization of the residual austenite in the heated part of the tool, which transformed to martensite during cooling in the air after removal from the tool. The effect of the cooling rate in the intercritical region was also not demonstrated in the paper by Kučerová et al. [24]. The same character of the microstructure was confirmed by metallographic analysis and hardness values, which ranged from 243 to 248 HV10.

In the case of longer tool dwell times (70 s and more) and therefore longer isothermal dwell times in the heated part of the tool, a significant difference in mechanical properties between the cooled and heated parts was observed. In the cooled parts, an ultimate tensile strength limit of around 1000 MPa and ductility in the range of 23 to 26% were achieved for the other processing parameters. A higher strength value than for the shortest dwell time was due to the higher proportion of martensite in the structure, which was also reflected by an increase in hardness, up to values of around 250 HV10. As far as the heated part is concerned, the length of the dwell time was evident. As the dwell time increased, there was a gradual decrease in ultimate strength from 914 MPa for a dwell time of 70 s to 818 MPa for a dwell time of 900 s. This decrease in strength was due to the loss of martensite in the structure and an increase in the proportion of bainite and retained austenite. This was also reflected by an increase in the ductility value, which here was between 35 and 36% (Figure 12). The hardness values in these regions decreased to values around 200 HV10 (Table 4).

**Figure 13:**
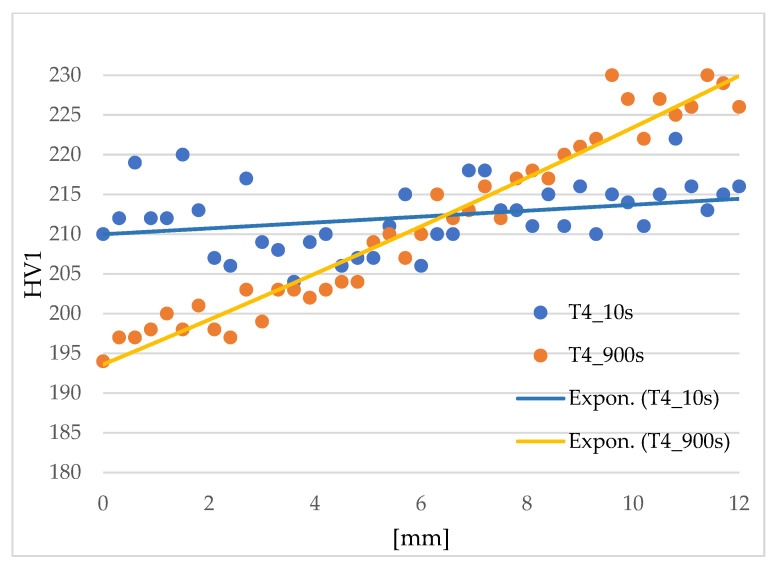
Hardness profile through the transition area of 20MnAl steel samples: 10s_T and 900s_T.

Since a change in mechanical properties was expected in the transition area, i.e., at the point of insulation between the cooled and heated part of the tool, the hardness profile over this area was measured for the 20MnAl material. The aim was to determine over how wide a region the change in properties occurs and whether there is a local step change that could cause problems. This hardness profile HV1 was measured over a total length of 12 mm with an indentation spacing of 0.3 mm. As can be seen in Figure 13, measured values were interleaved with exponential trend lines. The hardness results confirmed that for a 10 s sample there is almost no change in hardness across the region examined. For the 900 s sample, there was a gradual increase in hardness. The difference in hardness at the endpoints was approximately 30 HV1. There were no step changes in hardness in the material, which is especially important from the point of view of the use of the parts in terms of their behaviour under stress.

## 4. Discussion

**Figure 14:**
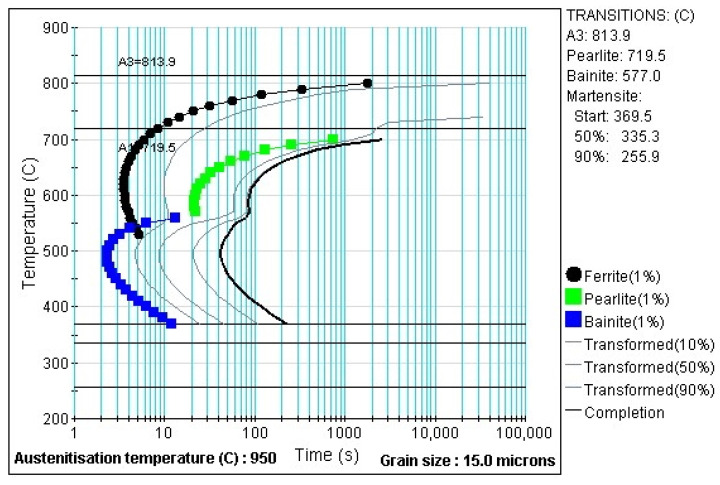
TTT diagram for 22MnB5 steel calculated in JMatPro software (version 12.1).

For 22MnB5 steel, it was found that in the combined press hardening tool in the cooled part of the omega profile, it was possible to obtain martensitic structures with bainite content and to reach strength limits exceeding 1400 MPa. These properties have previously been investigated e.g., in [3,4]. For this steel, a tool dwell time of 10 s at the heating point of 425 °C already showed a significant change in mechanical properties and microstructure (according to the diagram in Figure 5). The reduced cooling rate and the stopping of quenching at 425 °C led to the formation of a ferritic-pearlitic structure, which resulted in a decrease in ultimate tensile strength to 668 MPa. Longer dwell times in the heated tool from 70 to 210 s led to the promotion of bainite formation and only a small proportion of martensite was formed when cooling to room temperature after removal from the tool. The significant bainite formation at isothermal dwell was confirmed by the TTT diagram calculated for this steel, according to which bainite formation already occurs at dwell times around 3 s (Figure 14). These mixed structures showed higher strength limits of over 830 MPa than the ferritic-pearlitic structure and at the same time higher ductility values than the martensitic structures in the cooled part. The optimum combinations of the tailored properties were observed at 140 and 210 s.

**Figure 15:**
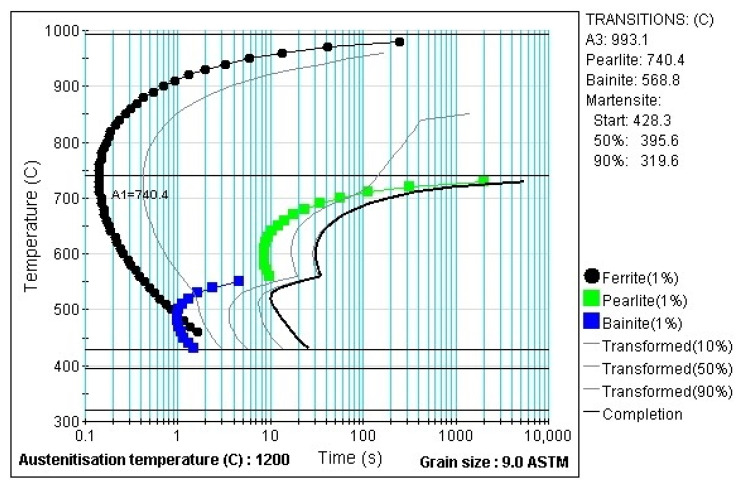
TTT diagram for 20MnAl steel calculated in JMatPro software.

The experimental steel 20MnAl is a multiphase steel that belongs to the group of steels using deformation-induced martensitic transformation to achieve the desired mechanical properties. In these steels, stability and the proportion of residual austenite in the microstructure are important [8,19,25]. As reported by B.C. De Cooman and X. Zhao, the evolution of the structure and the stability of the residual austenite are greatly influenced by the processing parameters, especially the temperature and the dwell time in the bainitic transformation region [26,27], which is carried out above the Ms temperature of the steel. Due to the carbon content and alloying elements, full austenitisation was not achieved at the selected heating temperature of 950 °C (Figure 15), which was chosen because of the sufficient stabilization by carbon of the formed austenite. Heating to high intercritical temperatures leads to the formation of a higher fraction of austenite with a lower carbon content, which is not sufficiently stable and thus does not contribute to the ductility enhancement [28]. Heating between Ac1 and Ac3 is also recommended by other authors. A higher proportion of residual austenite when heating to the middle of the interval between Ac1 and Ac3 was also found by S. Lee et al. [29,30] and L. Zhao [28].

For the experimental material 20MnAl, a fully martensitic structure was not achieved in the cooled part of the tool at any dwell time, which was due to the mixed martensitic-ferritic microstructure, with martensite forming in the fully or partially decomposed austenitic islands. When the omega profile in the tool was cooled down to room temperature, a strength limit of around 1000 MPa was obtained with a ductility of about 23 to 25%. Very similar results were obtained in [31], where a strength limit of about 930 MPa was obtained for 0.2C1.4Mn1.8Si steel. In the heated part of the tool, different dwell times at 425 °C were observed. As also reported by [32] the isothermal dwell in the bainitic transformation region affects both the proportion of bainite and the stability of the residual austenite. The ultimate strength dropped to values around 817 MPa at the longest dwell time, but the ductility increased up to 35%. Very similar results were also found by Kučerová et al. on steels of similar chemical composition, where an ultimate strength in the range of 805 to 859 MPa was achieved depending on the heating and deformation temperature [24,33].

Based on the obtained mechanical properties, dwell times of 70 or 140 s are recommended as suitable processing options to achieve the best properties in both the cooled and heated parts. With a dwell time of 140 s, a hardness of 256 ± 6 HV10 was achieved for the cooled part with a strength of 1008 MPa and a ductility of 23.5%, and for the heated part, a hardness of 207 ± 6 HV10 was achieved with a strength of 860 MPa and a ductility of almost 36%.

Future research will focus on testing other experimental materials and finding optimal parameters for hot stamping.

## 5. Conclusions

Press-hardening of two advanced high-strength steels was carried out to produce parts with tailored properties in one processing step, and save material and the weight of the parts while maintaining or improving their mechanical properties. In this paper, the ability of a special combined tool partially manufactured by 3D printing to produce sheets with different cooling rates was verified. The process was tested on the most frequently used 22MnB5 steel and then on experimental steel 20MnAl, where we obtained previously unreported results. For the purpose of comparison, the following processing parameters were used: heating the sheet for 15 min at 950 °C, then transferring it to the combined tool and holding it in the tool for 10, 70, 140, 210, and 900 s. The heated part was 425 °C and the cooled part was 20 °C.

For 22MnB5 steel, the cooling rate was found to have a large effect on the evolution of the microstructures and the resulting mechanical properties. Slower cooling in the heated part of the tool resulted in a significant decrease in ultimate tensile strength compared to the part formed in the cooled part of the tool. The decrease in strength was around 600 MPa with a tool dwell of 210 s. On the other hand, by removing the martensite from the structure, it was possible to increase the ductility by up to 25%. Steel 22MnB5 belongs to the group of boron steels, which are predestined by their chemical composition to form quench hardening structures. Good mechanical properties were already achieved at the shortest tool dwell time (i.e., 10 s) in the quenched region, which consisted of martensite and bainite and reached a hardness of 469 ± 7 HV10. Then, the heated part of the tool showed higher ductility values as the structure contained pearlite and ferrite and a hardness of 216 ± 4 HV10. These are results that are consistent with findings from other publications.

Compared to 22MnB5 steel, 20MnAl has lower strength limits but higher ductility values. The difference in hardness in the case of the 70 s sample is then approximately 60 HV10, with the microstructure containing proeutectoid ferrite, martensite, and a small proportion of retained austenite in the cooled part, with a hardness of 255 ± 5 HV10. Furthermore, in the heated part there is proeutectoid ferrite, bainite, and a large proportion of partially transformed islands of residual austenite to martensite with a hardness of 195 ± 3 HV10. For the multiphase 20MnAl steel, by slowing down the cooling rate in the heated part of the tool, a ductility of up to 36% could be achieved by promoting bainite formation and stabilizing the residual austenite with carbon. The difference between the heated and cooled parts of the omega profile was around 140 MPa for ultimate strength and around 10% for ductility.

## Figures and Tables

**Table 1:** Chemical composition of the materials (wt. %).

Material	C	Si	Mn	P	S	Al	Ti	Cr	B	Nb
22MnB5	0.25	0.4	1.35	0.023	0.01	0.08	0.03	0.25	0.004	-
20MnAl	0.2	0.51	1.75	0.007	0.001	1.39	-	0.19	-	0.056

**Table 2:** Parameters of experimental programme.

Material	Furnace Temperature (°C)	Soaking Time (min)	Tool Parts (°C)		Time in the Tool (s)
			Heated	Cooled	
22MnB5	950	15	c. 425	c. 20	10; 70; 140; 210; 900
20MnAl	950	15	c. 425	c. 20	10; 70; 140; 210; 900

**Table 3:** Hardness of 22MnB5 steel in different parts of the sheet.

Material	Sample	HV10	Sample	HV10	Sample	HV10	Sample	HV10	Sample	HV10
22MnB5	10s_C	469 ± 7	70s_C	386 ± 6	140s_C	420 ± 3	210s_C	481 ± 7	900s_C	453 ± 1
	10s_T	254 ± 2	70s_T	357 ± 12	140s_T	342 ± 8	210s_T	279 ± 2	900s_T	245 ± 2
	10s_H	216 ± 4	70s_H	255 ± 2	140s_H	259 ± 4	210s_H	265 ± 5	900s_H	219 ± 2

**Table 4:** Hardness of 20MnAl steel in different parts of the sheet.

Material	Sample	HV10	Sample	HV10	Sample	HV10	Sample	HV10	Sample	HV10
20MnAl	10s_C	243 ± 3	70s_C	255 ± 5	140s_C	256 ± 6	210s_C	249 ± 1	900s_C	240 ± 2
	10s_T	232 ± 2	70s_T	223 ± 2	140s_T	207 ± 6	210s_T	217 ± 4	900s_T	200 ± 6
	10s_H	248 ± 2	70s_H	195 ± 3	140s_H	210 ± 4	210s_H	205 ± 1	900s_H	198 ± 1

## Data Availability

The raw data are not publicly available due to ongoing research.
